# Sensitivity and specificity of self-reported psychiatric diagnoses amongst patients treated for opioid use disorder

**DOI:** 10.1186/s12888-021-03489-4

**Published:** 2021-10-21

**Authors:** Kelly Leung, Emily Xu, Tea Rosic, Andrew Worster, Lehana Thabane, Zainab Samaan

**Affiliations:** 1 Michael G. DeGroote School of Medicine, McMaster University, Hamilton, Ontario Canada; 2 Department of Psychiatry and Behavioral Neurosciences, McMaster University, Hamilton, Ontario Canada; 3 Department of Health Research Methods, Evidence, and Impact, McMaster University, Hamilton, Ontario Canada; 4 Biostatistics Unit, Research Institute at St Joseph’s Healthcare, Hamilton, Ontario Canada; 5 Departments of Pediatrics/Anesthesia, McMaster University, Hamilton, Ontario Canada

**Keywords:** Opioid use disorder, Psychiatric diagnoses, Self-reported diagnoses

## Abstract

**Background:**

Patients with opioid use disorder (OUD) frequently present with comorbid psychiatric illnesses which have significant implications for their treatment outcomes. Notably, these are often identified by self-report. Our study examined the sensitivity and specificity of self-reported psychiatric diagnoses against a structured diagnostic interview in a cohort of patients receiving outpatient pharmacological treatment for OUD.

**Methods:**

Using cross-sectional data from adults receiving outpatient opioid agonist treatment for OUD in clinics across Ontario, Canada, we compared participants’ self-reported psychiatric diagnoses with those identified by the Mini Neuropsychiatric Interview (MINI) Version 6.0 administered at the time of study entry. Sensitivity and specificity were calculated for self-report of psychiatric diagnoses.

**Results:**

Amongst a sample of 683 participants, 24% (*n* = 162) reported having a comorbid psychiatric disorder. Only 104 of these 162 individuals (64%) reporting a comorbidity met criteria for a psychiatric disorder as per the MINI; meanwhile, 304 (75%) participants who self-reported no psychiatric comorbidity were in fact identified to meet MINI criteria for a psychiatric disorder. The sensitivity and specificity for any self-reported psychiatric diagnoses were 25.5% (95% CI 21.3, 30.0) and 78.9% (95% CI 73.6, 83.6), respectively.

**Conclusions:**

Our findings raise questions about the utility of self-reported psychiatric comorbidity in patients with OUD, particularly in the context of low sensitivity of self-reported diagnoses. Several factors may contribute to this including remittance and relapse of some psychiatric illnesses, underdiagnosis, and the challenge of differentiating psychiatric and substance-induced disorders. These findings highlight that other methods should be considered in order to identify comorbid psychiatric disorders in patients with OUD.

**Supplementary Information:**

The online version contains supplementary material available at 10.1186/s12888-021-03489-4.

## Background

The opioid crisis continues to be a major public health problem worldwide [1–4]. In 2017, it was estimated that 40.5 million people globally were dependent on opioids, although trends varied between countries [1]. The burden of opioid use and dependence is significantly more prevalent in North America compared to other parts of the world [1]. In Canada, overdose deaths and hospitalizations related to opioid use disorder (OUD) have remained high despite increasing awareness and intervention [5, 6]. In the United States, opioids are involved in the majority of deaths related to drug overdose, which rose to an all-time high in 2019 with 49,860 overdose deaths involving opioids [7–9]. The impacts of the COVID-19 pandemic have additionally exacerbated pre-existing issues relating to access to appropriate treatment and interventions, ensuring that the opioid crisis will remain an ongoing concern for patients and families, healthcare workers, and policymakers alike [9].

Patients with substance use disorders (SUD) experience comorbid psychiatric disorders, such as depression and anxiety, at rates higher than the general population [10]. Canadian data suggest that the 12-month prevalence of psychiatric comorbidity is 5 to 6 times higher among individuals with substance dependence compared to the general population (OR 5.29, 95% CI 3.90, 7.17) [10]. Additionally, patients with psychiatric disorders report higher rates of substance use problems and are approximately twice as likely to have SUDs [10, 11]. This comorbidity is exacerbated in patients with OUD who appear to have even higher lifetime prevalence of psychiatric disorders compared to those with other SUDs and the general population; lifetime rates are generally greater than 40%, although there is considerable variability in reported rates across studies [12]. We have previously found that among patients receiving methadone maintenance treatment for OUD, approximately 80% of participants had a comorbid psychiatric disorder, with 43% having an anxiety disorder, and 41% having a mood disorder [11]. Psychiatric disorders are similarly associated with an increased risk of developing OUD in individuals who are prescribed opioids for pain (OR 1.46, 95% CI 1.12, 1.91) [13].

The importance of understanding a patient’s psychiatric comorbidities in the context of their OUD should not be understated. When assessing patients, it may be challenging to determine whether their symptoms are due to a psychiatric comorbidity, or secondary to substance use [14]. These comorbidities also must be considered in the context of treatment. For patients with OUD, psychiatric comorbidities have been associated with worse clinical outcomes. A study examining retention in buprenorphine treatment found the presence of any comorbid mental illness to have a hazard ratio of 1.05 (95% CI 1.01, 1.10) for treatment discontinuation at three years [15] while inpatient mental health treatment was associated with a hazard ratio of 1.2 (95% CI 1.19, 1.30) for treatment discontinuation [16]. Similarly, psychiatric comorbidity conferred higher hazard for attrition from methadone treatment (hazard ratio 1.20, 95% CI 1.06, 1.35) [15]. In studying the impact of psychological symptoms in OUD treatment outcomes, we have previously found that psychological symptoms were associated with higher percentages of opioid-positive drug screens (*B* = 0.02, 95% CI 0.01–0.02 = 3) and non-opioid-positive drug screens (*B* = 1.92, 95% CI 0.89, 2.95) [14]. The prevalence of depression and anxiety disorders in opioid-related overdoses reaches rates greater than 30% [17], and severe depression (OR 2.46, 95% CI 1.24, 4.89), post-traumatic stress disorder (PTSD) (OR 2.77, 95% CI 1.37, 5.60), and psychosis (OR 2.39, 95% CI 1.10, 5.15) are significantly associated with opioid overdoses [18]. In a study looking at factors associated with mortality among heroin users, a history of psychiatric treatment was associated with a hazard ratio of 6.21 (95% CI 2.52, 15.33) for all-cause mortality [19].

Psychiatric comorbidity is also associated with lower reported quality of life for patients with OUD [20]. Epidemiological data on patient outcomes, in addition to other clinical and systems-level research, have reinforced the need for concurrent disorders treatment that integrates treatment for psychiatric disorders and addictions [10, 21].

Most commonly in substance use treatment programs, the record of historical mental health diagnoses is based on patient self-report [22]. The utility of self-reported psychiatric diagnoses within patients with SUDs was examined by Neumann and colleagues [22] who found self-reported diagnoses of bipolar disorder and depression were often not confirmed by current clinical measures, and many participants had undiagnosed psychiatric disorders. In light of these findings and a clinical reliance on patient self-report, the objective of this study was to examine the sensitivity and specificity of patient-reported psychiatric diagnoses in a cohort of patients receiving outpatient pharmacological treatment for OUD.

## Methods

Data were collected in the GENetics of Opioid Addiction (GENOA) prospective cohort study conducted in Ontario, Canada. The objective of the GENOA study was to examine biological and social factors affecting treatment outcome and course for patients with OUD [23]. Participants were recruited from 20 outpatient opioid agonist treatment clinics between 2011 and 2017. All clinics were run by the same management teams and followed the same treatment protocols. Inclusion criteria required participants to be a minimum of 18 years of age, to meet diagnostic criteria for OUD as per DSM-IV criteria, [24] and to be enrolled in opioid agonist therapy (primarily methadone). At the time that this study was conducted, buprenorphine-naloxone was not covered through the Ontario provincial drug plan, thus greatly limiting its access, and making methadone the primary opioid substitution treatment available. No restrictions were placed on participants’ duration in treatment; therefore, individuals were enrolled in treatment for different lengths of time at the time of study entry. This study was conducted in accordance with the Declaration of Helsinki Ethical Principles for Medical Research Involving Human Subjects. Ethics approval was obtained from the Hamilton Integrated Research Ethics Board (project ID 11–056) and all participants provided informed consent. We report the present study following the Strengthening the Reporting of Observational Studies in Epidemiology (STROBE) guidelines [25].

At study intake, all participants completed extensive baseline assessments performed by trained interviewers, including assessment of sociodemographic and clinical information (including opioid agonist treatment history). All participants were asked to self-report their medical history, with the open-ended question: “Do you have any medical conditions or health problems?” Responses to this question were recorded in an open-ended text field and later summarized into diagnostic categories by two study authors (KL and EX) in duplicate for consistency and accuracy.

Self-reported psychiatric diagnoses were compared against a diagnostic interview using the Mini Neuropsychiatric Interview (MINI) version 6.0 [26] a standardized and structured tool widely used in research and clinical settings [24]. The MINI has been validated against the Structured Clinical Interview for Diagnostic and Statistical Manual of Mental Disorders (SCID) and the Composite International Diagnostic Interview (CIDI) [26]. In comparison to the SCID, the sensitivity of the MINI was 70% or greater for 19 out of 22 diagnoses evaluated by the SCID while the specificity was 85% or higher for all diagnoses. When compared to the CIDI, the MINI had a sensitivity of 70% or greater for 7 out of 11 diagnoses captured by the CIDI and had a specificity of 70% or greater for all diagnoses. The MINI had excellent interrater reliability with 70% of interrater kappa values 0.90 or above. The retest reliability of the MINI was good with 61% of the retest kappa values being above 0.75. Additionally, the agreement between diagnoses made by general practitioners using the MINI and diagnoses from psychiatrists was 85% [26]..

The first 690 participants consecutively recruited into the study were administered the MINI version 6.0 [26]. Use of the MINI was subsequently discontinued due to time burden of administration. The MINI interview encompasses assessments of mood disorders (major depression and bipolar disorder), anxiety disorders (panic disorder, agoraphobia, social anxiety disorder, generalized anxiety disorder), obsessive-compulsive disorder (OCD), PTSD, psychotic disorders (schizophrenia, schizoaffective disorder, schizophreniform disorder, brief psychotic disorder, substance induced psychotic disorder, and psychotic disorder not otherwise specified), and eating disorders (anorexia nervosa and bulimia nervosa). In order to reasonably compare self-reported psychiatric diagnoses with diagnoses identified by structured diagnostic interview, we only considered relevant the diagnoses assessed for in the MINI (for example, attention deficit hyperactivity disorder is not included in the MINI and was therefore excluded from self-report). Antisocial personality disorder is examined in the MINI, however this diagnosis was not self-reported by any of the study participants and was therefore excluded from the present analyses. No other personality disorders are identified using the MINI.

We used descriptive statistics to summarize participant demographic and clinical characteristics, using mean values with standard deviation (SD) or median values with interquartile range (IQR) for continuous variables, and frequencies for categorical variables. Sensitivity and specificity values were calculated for each of the following disorders: any psychiatric disorder, depression, bipolar disorder, anxiety disorder, OCD, PTSD, psychotic disorder, and eating disorder. All analyses were conducted using Stata version 15.1.

## Results

**Fig. 1:**
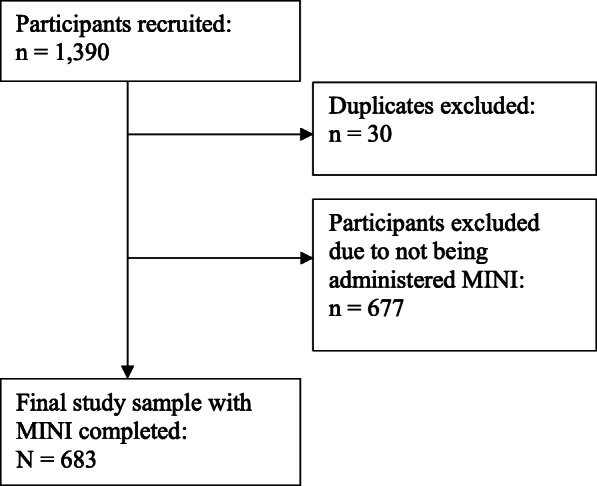
Study Flow Diagram

Altogether, 1390 participants were recruited into the GENOA study, and upon exclusion of participants recruited in duplicate (*n* = 30) and participants who did not receive the MINI diagnostic interview (*n* = 677), our final study sample consisted of 683 participants (Fig. 1. Study flow diagram). We summarize participant characteristics in Table 1: the mean age was 39.1 years (SD = 11.4) and males composed 58% of the cohort (*n* = 395).

**Table 1:** Participant characteristics at study entry (*N* = 683)

Characteristic	Total sample *N* = 683	Self-reported psychiatric comorbidity *n* = 162 (23.7%)	No self-reported psychiatric comorbidity *n* = 521 (76.3%)
Age (years); mean (SD)	39.1 (11.4)	37.8 (11.6)	39.4 (11.3)
Sex; n (%)			
Males	395 (57.8%)	94 (58.0%)	301 (57.8%)
Females	288 (42.2%)	68 (42.0%)	220 (42.2%)
Unemployment; *n* (%)	450 (65.9%)	108 (66.7%)	342 (65.5%)
Age at first opioid use (years); mean (SD)	25.5 (8.8)	25.2 (8.7)	25.6 (8.8)
Years in treatment; median (Q1, Q3)	3 (1, 6)	2 (1, 5)	3 (1, 7)
Methadone dose (mg/d); mean (SD)	77.0 (46.5)	71.8 (39.8)	78.7 (48.3)
Opioid abstinence at study entry; *n* (%)	335 (49.1)	71 (43.8)	264 (50.7)
Percentage (%) of opioid-positive drug screens at study entry if non-abstinent; median (Q1, Q3)	25 (10, 58.2)	29.6 (12, 64.3)	23.5 (9.1, 55.6)

*SD* Standard Deviation, *Q1* Quartile 1, *Q3* Quartile 3

The unemployment rate was 66% (*n* = 450). The reported mean age at first opioid use was 25.5 years (SD = 8.8) and the median years spent in treatment was 3 (Q1, Q3 = 1, 6). The mean methadone dose for the participants was 77 mg/day (SD = 46.5). Three participants in this study sample were prescribed buprenorphine-naloxone (0.4%) at the time of recruitment. At study entry, the percentage of participants that reported being abstinent from opioids was 49% (*n* = 335). The median percentage of opioid-positive drug screens in participants who reported being non-abstinent at the beginning of the study was 25% (Q1, Q3 = 10, 58.2).

Amongst all study participants, 104 (15.2%) reported a diagnosis of depression, 15 (2.2%) reported a diagnosis of bipolar disorder, 102 (14.9%) reported a diagnosis of an anxiety disorder, and 27 (4.0%) reported a diagnosis of PTSD. Less frequently reported diagnoses were OCD, psychotic disorders, and eating disorders. In total, 162 (23.7%) participants self-reported a history of psychiatric comorbidity (Table 2).

**Table 2:** Psychiatric comorbidity identified within the study sample (*N* = 683)

Psychiatric comorbidity	Self-reported	MINI-Diagnosed
Any psychiatric disorder; *n* (%)	162 (23.7%)	408 (59.7%)
Depression; *n* (%)	104 (15.2%)	190 (27.8%)
Bipolar disorder; *n* (%)	15 (2.2%)	90 (13.2%)
Anxiety disorder; *n* (%)	102 (14.9%)	229 (33.5%)
PTSD; *n* (%)	27 (4.0%)	72 (10.5%)
OCD; *n* (%)	4 (0.6%)	76 (11.1%)
Psychotic disorder; *n* (%)	4 (0.6%)	25 (3.7%)
Eating disorder; *n* (%)	1 (0.2%)	8 (1.2%)

*MINI* Mini-International Neuropsychiatric Interview, *PTSD* Post-traumatic Stress Disorder, *OCD* Obsessive Compulsive Disorder

The sensitivity and specificity of self-reported psychiatric diagnoses are reported in Table 3.

**Table 3:** Sensitivity and specificity of self-reported psychiatric disorders (*N* = 683)

	Sensitivity (95% CI)	Specificity (95% CI)
Any psychiatric disorder	25.5% (21.3, 30.0)	78.9% (73.6, 83.6)
Any depressive disorder	17.9% (12.7, 24.1)	85.8% (82.4, 88.8)
Bipolar disorder	0% (0, 4.0)	97.5% (95.9, 98.6)
Any anxiety disorder	14.8% (10.5, 20.1)	85% (81,4, 88.2)
PTSD	4.2% (0.8, 11.7)	96.1% (94.2, 97.5)
OCD	0% (0, 4.7)	99.3% (98.3, 99.8)
Psychotic disorder	0% (0, 13.7)	99.4% (98.5, 99.8)
Any eating disorder	0% (0, 36.9)	99.9% (99.2, 99.996)

*CI* Confidence Interval, *PTSD* Post-traumatic Stress Disorder, *OCD* Obsessive Compulsive Disorder

Of the 162 participants who self-reported any psychiatric comorbidity, 104 met criteria for a comorbidity as per the MINI and 58 did not. Furthermore, 304 participants who reported no psychiatric comorbidity were identified to meet MINI criteria for a psychiatric disorder. The sensitivity and specificity for any self-reported psychiatric diagnoses was 25.5% (95% CI 21.3, 30.0) and 78.9% (95% CI 73.6, 83.6), respectively.

The sensitivity and specificity for self-reported depression was 17.9% (95% CI 12.7, 24.1) and 85.8% (95% CI 82.4, 88.8), respectively; meanwhile the sensitivity and specificity for self-reported anxiety disorders was 14.8% (95% CI 10.5, 20.1) and 85% (95% CI 81.4, 88.2), respectively; and the sensitivity and specificity for self-reported PTSD was 4.2% (95% CI 0.8, 11.7) and 96.1% (95% CI 94.2, 97.5), respectively. The sensitivity for each of self-reported bipolar disorder, OCD, psychotic disorder, and eating disorders was 0% (95% CI for bipolar disorder 0, 4.0; 95% CI for OCD 0, 4.7; 95% CI for psychotic disorder 0, 13.7; 95% CI for eating disorder 0, 36.9). The specificity was 97.5% for self-reported bipolar disorder (95% CI 95.9, 98.6), 99.3% for OCD (95% CI 98.3, 99.8), 99.4% for psychotic disorder (95% CI 98.5, 99.8), and 99.9% for eating disorders (95% CI 99.2, 99.996). There were many new diagnoses identified using the MINI including 156 diagnoses of depression, 195 diagnoses of anxiety, 69 diagnoses of PTSD, 90 diagnoses of bipolar disorder, 76 diagnoses of OCD, 25 diagnoses of psychotic disorders, and 8 diagnoses of eating disorders.

## Discussion

Overall, we found that the sensitivity of self-reported psychiatric comorbidity was low, when compared to the MINI, particularly for self-reported depression, anxiety disorder, PTSD, and overall psychiatric diagnoses. In addition, none of the patients with a self-reported history of bipolar disorder, OCD, psychotic disorder, or eating disorder met the criteria for a current diagnosis of each disorder. Many new diagnoses were made in participants who did not report a history of these disorders - including but not limited to depression, anxiety, PTSD, and bipolar disorder. Although the specificity of self-report was higher for certain diagnoses (including anxiety disorder and PTSD), the overall low sensitivity illustrates that self-report did not accurately capture the patients’ present clinical picture. Thus, the utility of self-reported psychiatric comorbidity is in question.

There are several potential explanations for the low sensitivity of self-reported psychiatric disorders. Psychiatric disorders can be episodic in nature, with recurrent symptoms that cycle through relapsing-remitting courses. Participants who reported a historical diagnosis may no longer be experiencing symptoms and no longer meet the criteria for that disorder at the time of assessment. Similarly, participants for whom new psychiatric diagnoses were made may not have previously met criteria for the respective diagnoses. In addition, psychiatric disorders are likely under-diagnosed in the OUD population as evidenced by the under-diagnosis of concurrent disorders as a whole [27]. There may be poor health literacy within the OUD population which can negatively impact an individual’s ability to recognize symptoms of a mental disorder and then communicate these issues to a healthcare provider, contributing to underdiagnosis [28]. Furthermore, treatment for mental health and substance abuse disorders continues to be offered separately, despite the evidence supporting concurrent treatment, and between navigating both systems diagnoses may be missed.

Most studies examining the sensitivity and specificity of self-reported psychiatric diagnoses do not include participants with SUDs. For instance, studies have found moderate to high agreement between self-reported and clinically-diagnosed depression in groups of university graduates and adults being followed for osteoporosis [29, 30]. Conversely, a self-report tool used to screen members of the United States Armed Forces for deployment was found to have low validity in identifying individuals with diagnosed mental health disorders [31]. Much of the related literature in the patients with SUDs examines self-reported substance use, rather than psychiatric comorbidity [32]. Neumann et al. compared self-reported historical psychiatric diagnoses with current clinical diagnoses assessed using the MINI screen, MINI Plus 500 structured interview, and Personality Diagnostics Questionnaire-4th edition. They found that self-reported diagnoses such as bipolar disorder and depression were often not verified by current clinical measures [22]. In addition, many patients had undiagnosed personality disorders, depression, and anxiety disorders [22]. To our knowledge, no other studies have examined the sensitivity and specificity of self-reported psychiatric diagnoses in SUD or OUD populations.

As discussed, individuals with OUD have higher rates of co-occurring psychiatric comorbidities compared to the general population, with rates reaching greater than 40% across their lifetime, [12] and these comorbidities are associated with increased psychiatric distress, rates of SUD, and poorer response to substance use treatment [10, 11, 33]. Despite this, the evidence surrounding the efficacy of psychiatric treatment in these individuals remains equivocal. Literature on pharmacotherapy (mostly consisting of placebo-controlled medication trials) has shown mixed results, with many studies showing no difference from placebo in reducing psychiatric symptoms or substance use [33]. With respect to psychosocial interventions, a Cochrane review found that patients receiving psychosocial interventions in addition to OAT did not show improved outcomes compared to individuals receiving OAT alone; whereas another systematic review did support their efficacy to improve clinical outcomes [34]. There is emerging evidence that some of these inconsistent findings may be indicative of other variables impacting patient responses to psychiatric treatment, including ongoing substance use and complex psychosocial such as housing and employment [9, 33, 35].

The varied, interlinked, and complex needs of individuals with OUD (and other SUDs) have led to a movement towards concurrent treatment [10, 21]. While the evidence for how psychiatric treatment impacts outcomes for patients with SUDs is conflicting, there have been encouraging results from literature evaluating integrated care for both psychiatric and SUDs. In patients receiving both OAT and psychiatric care, those who received on-site and integrated care of both had improved psychiatric outcomes compared to those who received their psychiatric care off-site and non-integrated with their substance use treatment; however no difference was found in outcomes related to substance use [36]. This remains an area requiring further research to elucidate how to best support these clinically complex patients and improve their outcomes.

For patients experiencing SUDs, clinicians also face challenges differentiating between psychiatric disorders and substance-induced disorders as both intoxication and withdrawal from opioids can mimic the symptomatology seen in mood, anxiety, and psychotic disorders [12, 37, 38]. The difficulty in delineating between psychological symptoms as a result of a primary psychiatric disorder versus secondary to SUD (including OUD) makes diagnosing mental illness in this population a complex challenge [12, 37]. In fact, due to the overlap in symptoms between symptom intoxication/withdrawal and affective disorders, Quello et al. advise that definitive assessments of psychiatric diagnoses should be postponed until individuals have achieved a reasonable period of abstinence, although the length of this period varies [39]. Instead they suggest using screening methods to identify possible mood disorders, with associated follow-up after abstinence for definitive diagnosis [39]. Furthermore, individuals may not recall, choose not to disclose, or disagree with a previous psychiatric diagnosis; in addition to self-diagnosing themselves incorrectly. This could occur for various reasons relating to the social stigma of substance use, the impacts of chronic substance use or untreated psychiatric disorders on memory, lack of access to healthcare for appropriate psychiatric assessment, among others.

Our results highlight the need for improved methods to ascertain comorbid psychiatric disorders in the OUD population during treatment. While the specificities of many psychiatric disorders were high, the sensitivities remained poor. That several patients in this study received new diagnoses additionally highlights the issue of under-diagnosis of psychiatric comorbidities in this population, and the need for better ways to identify them [27]. Potential alternatives to patient self-report of psychiatric diagnoses may include structured interviews or questionnaires. In the setting of an addiction treatment clinic, time and resource limitations, and clinical expertise influence the ability to conduct diagnostic interviews and comprehensively assess psychiatric symptomatology. The GENOA study, from which our data were obtained, initially used the MINI structured diagnostic interview, which takes approximately 20 min to administer and use of this tool was later discontinued due to the time burden of administration on participants who did not like the additional 20 min it took to complete the interview. However, the MINI is one of the quickest diagnostic tools used to assess for psychiatric disorder, and other standardized assessments such as the CIDI [40] or the SCID [41] require more time to administer compared to the MINI [42].

Since diagnostic tools require significant time resources, an alternative option would be to administer screening tools that take less time to complete in order to identify the patients who may benefit from a more detailed diagnostic interview. This would be particularly pertinent in relation to the low sensitivities of our results as a way to provide diagnostic clarification when patients self-report psychiatric concerns. Examples of screening tools which require less than 10 min to complete include the Brief Symptom Inventory, [43] Modified MINI screen, [44] and the General Health Questionnaire [45]. The Dual Diagnosis Screening Instrument [46] and Mental Health Screening Form-III [47] are both screening tools that have been developed specifically for the detection of psychiatric comorbidity in patients with SUDs. Another potential solution would be to have a psychiatrist on-site who would be able to administer assessments for psychiatric disorders, which has also been shown to increase patient engagement with psychiatric services [48].

A limitation of this study was the way self-reported psychiatric disorders were elicited, with an open-ended question inquiring about any medical or health problems. This question may have been understood as asking for solely non-psychiatric medical problems or only for current issues that the patient is experiencing, not past diagnoses that are now in remittance. A question that explicitly asks around a history of mental health disorders both past and present would be less susceptible to misinterpretation. Additionally, the MINI does not assess for personality disorders, which are heavily implicated in SUD, [49] or attention deficit hyperactivity disorder. Thus, analysis regarding the sensitivity and specificity of self-report for these disorders is unavailable. Importantly, there is an under-reporting and under-recognition of psychiatric comorbidities in the context of OUD despite the demonstrated impact of such comorbidity on treatment outcomes. Health care providers need to be aware of the prevalence of such disorders in this population and policies regarding systematic screening of patients attending treatment for SUDs for comorbid psychiatric conditions are needed. The choice of a screening tool or clinical assessment that is feasible to use must balance accuracy, effort, and scalability. It is also possible that for some patients, poor mental health literacy in the SUD population likely further exacerbates the issue of self-recognition of psychiatric symptoms and subsequently seeking help with diagnosis.

## Conclusion

The sensitivity and specificity of self-reported psychiatric comorbidity in patients with OUD was found to be low when compared to standardized, structured, diagnostic interviews. The remitting-relapsing nature of psychiatric disorders, under-diagnosis, the difficulty in differentiating independent psychiatric disorders from substance-induced disorders, and patient factors likely contributed to these findings. These findings highlight that other methods should implemented in order to identify comorbid psychiatric disorders in patients with OUD.

## Supplementary Information

**Additional file 1: Appendix A.** Paired contingency tables comparing results of self-reported diagnosis to MINI diagnosis.

## Data Availability

The data that support the findings of this study are available from the corresponding author, ZS, upon reasonable request.

## Abbreviations

OUD: Opioid use disorder
MINI: Mini International Neuropsychiatric Interview
SUD: Substance use disorder
GENOA: GENetics of Opioid Addiction
SCID: Structured Clinical Interview for Diagnostic and Statistical Manual of Mental Disorders
CIDI: Composite International Diagnostic Interview
OCD: Obsessive compulsive disorder
PTSD: Post-traumatic stress disorder
