# Supplementary material for: Sensitivity and specificity of self-reported psychiatric diagnoses amongst patients treated for opioid use disorder
Source: BMC Psychiatry. 2021 Oct 21;21:520. doi: 10.1186/s12888-021-03489-4 (PMC8530009; doi:10.1186/s12888-021-03489-4)
Supplement: Supplementary file 1 — Additional file 1: Appendix A. Paired contingency tables comparing results of self-reported diagnosis to MINI diagnosis. [file 12888_2021_3489_MOESM1_ESM.pdf]

Supplementary Information

Appendix A. Paired contingency tables comparing results of self-reported diagnosis to Mini International Neuropsychiatric Interview diagnosis.

**Any psychiatric disorder**		**MINI Interview**	
		Positive	Negative
**Self-report**	Positive	104	58
	Negative	304	217

**Any depressive disorder**		**MINI Interview**	
		Positive	Negative
**Self-report**	Positive	34	70
	Negative	156	423

**Bipolar disorder**		**MINI Interview**	
		Positive	Negative
**Self-report**	Positive	0	15
	Negative	90	578

**Any anxiety disorder**		**MINI Interview**	
		Positive	Negative
**Self-report**	Positive	34	68
	Negative	195	386

**Post-traumatic stress disorder**		**MINI Interview**	
		Positive	Negative
**Self-report**	Positive	3	24
	Negative	69	587

**Obsessive compulsive disorder**		**MINI Interview**	
		Positive	Negative
**Self-report**	Positive	0	4
	Negative	76	603

**Any psychotic disorder**		**MINI Interview**	
		Positive	Negative
**Self-report**	Positive	0	4
	Negative	25	654

**Any eating disorder**		**MINI Interview**	
		Positive	Negative
**Self-report**	Positive	0	1
	Negative	8	674
